# Do mass media campaigns improve physical activity? a systematic review and meta-analysis

**DOI:** 10.1186/0778-7367-71-20

**Published:** 2013-08-02

**Authors:** Ajibola I Abioye, Kaveh Hajifathalian, Goodarz Danaei

**Affiliations:** 1 Department of Global Health and Population, Harvard School of Public Health, 665 Huntington Avenue, Bldg. 1, Room 1107, Boston, MA, USA; 2 Department of Epidemiology, Harvard School of Public Health, Boston, MA, USA

**Keywords:** Physical activity, Mass media, Systematic review, Meta-analysis, Prospective studies, Sedentary behavior

## Abstract

**Background:**

Mass media campaigns are frequently used to influence the health behaviors of various populations. There are currently no quantitative meta-analyses of the effect of mass media campaigns on physical activity in adults.

**Methods:**

We searched six electronic databases from their inception to August 2012 and selected prospective studies that evaluated the effect of mass media campaigns on physical activity in adults. We excluded studies that did not have a proper control group or did not report the uncertainties of the effect estimates. Two reviewers independently screened the title/abstracts and full articles. We used random-effects models to pool effect estimates across studies for 3 selected outcomes.

**Results:**

Nine prospective cohorts and before-after studies that followed-up 27,601 people over 8 weeks to 3 years met the inclusion criteria. Based on the pooled results from these studies, mass media campaigns had a significant effect on promoting moderate intensity walking (pooled relative risk (RR) from 3 studies=1.53, 95% Confidence Interval: 1.25 to 1.87), but did not help participants achieve sufficient levels of physical activity [4 studies pooled RR=1.02, 95% CI: 0.91 to 1.14)]. The apparent effect of media campaigns on reducing sedentary behavior (pooled RR=1.15, 95% CI: 1.03 to 1.30) was lost when a relatively low-quality study with large effects was excluded in a sensitivity analysis. In subgroup analyses, campaigns that promoted physical activity as a ‘social norm’ seemed to be more effective in reducing sedentary behavior.

**Conclusion:**

Mass media campaigns may promote walking but may not reduce sedentary behavior or lead to achieving recommended levels of overall physical activity. Further research is warranted on different campaign types and in low- and middle- income countries.

## Background

The global burden of non-communicable diseases (NCDs) is rising rapidly due to ageing, poor dietary habits, harmful use of alcohol, obesity, smoking and insufficient physical activity [1]. Globally, insufficient physical activity is the 10^th^ leading risk factor for disease burden causing 3.2 million deaths and accounting for 31% of burden of disease from ischemic heart disease [1]. However, a large proportion of the adults in both developed and developing countries fail to achieve the recommended levels of physical activity [2]. Several systematic reviews have evaluated the effectiveness of different types of population- and individual-level interventions to improve physical activity [3-6]. However, four prior reviews that examined the effectiveness of mass media campaigns only included articles published in English, or over a short period of time [7,8] and did not estimate the pooled effects due to heterogeneity in the effect measures reported in the original studies [7-10].

A quantitative estimate of the effect of mass media campaigns in promoting physical activity is required to compare the efficacy of alternative interventions, conduct cost-effectiveness analyses and make policy recommendations to prevent the epidemic of non-communicable diseases in developed and developing countries [11]. The goal of this study was to conduct a systematic review and meta-analysis of the effect of mass media campaigns on physical activity.

## Methods

### Search strategy

We identified original research articles in Medline (through PubMed), EMBASE (Elsevier), Cochrane Library, CINAHL, PsycINFO and Web of Science, and reviewed Google Scholar for additional relevant papers. Database searches were concluded in August 2012. We searched PubMed using a number of Medical Subject Headings (MeSH) terms representing health promotion, mass media and physical activity (Table 1). We restricted the search to studies of adults (≥19 years old) but did not restrict by year of publication or language. We searched EMBASE using similar search terms and restrictions and checked the reference list of systematic reviews for additional articles. Two authors (A.A. and K.H.) independently screened the titles and abstracts for eligibility and subsequently examined the full texts of the articles. Any discrepancies were resolved in consultation with the third author (G.D.).

**Table 1:** Medical literature database search for study selection

**Database**	**Search query**
PUBMED	("Health Promotion" [Mesh] OR "Health Behavior" [Mesh] OR "cohort studies" [Mesh] OR "Program Evaluation" [Mesh]) AND ("Mass Media" [Mesh] OR "television" [tiab] OR "radio" [tiab] OR "newspaper" [tiab] OR "magazine" [tiab]) AND ("Motor Activity" [Mesh] OR "sports" [Mesh] OR "physical fitness" [Mesh] OR "physical exertion" [Mesh] OR "physical activity" [All Fields] OR "exercise" [All Fields] OR "walking" [Mesh]) AND ("humans" [MeSH Terms] AND "adult" [MeSH Terms])
EMBASE	'health promotion'/exp AND ('mass medium'/exp OR 'television'/exp) AND ('physical activity'/exp OR 'exercise'/exp OR 'sport'/exp) AND ([adult]/lim OR [aged]/lim)
CINAHL	‘health promotion’ AND [‘mass media’ OR ‘television’ OR ‘radio’ OR ‘newspaper’ OR ‘magazine’] AND [‘physical activity’ OR ‘exercise’ OR ‘walking’ OR ‘sport’]
PsycInfo	‘health promotion’ AND [‘mass media’ OR ‘television’ OR ‘radio’ OR ‘newspaper’ OR ‘magazine’] AND [‘physical activity’ OR ‘exercise’ OR ‘walking’ OR ‘sport’]

In both rounds of title/abstract and full text review, we excluded studies that did not evaluate the effect of mass media campaigns on physical activity. These included case-reports, cross-sectional studies, baseline-only or terminal-only surveys, and studies including only information on awareness, attitude or knowledge. Studies reporting mass media campaigns that were implemented simultaneously with other interventions were also excluded. If several articles reported results from the same study with the same effect measure, we included the more recent report unless an earlier report provided the relevant effect measures or had a higher quality. Studies that did not report measures of uncertainty for the effect estimates were excluded.

### Data extraction

We extracted data on characteristics of the study population, including sample size, sampling method, eligibility criteria, mean or median follow-up time, response rate, age, sex and body mass index (BMI). We also extracted data on campaign characteristics including duration, scope, type of media, frequency and coverage; whether the campaigns were based on behavior change theory, prior research, or consultation with experts; use of risk message, shocking effects, social norm or celebrities. Coverage refers to the estimated proportion of the target population that is reported to have been reached by the media campaign. Point estimates of effect measures (odds ratios and difference measures) and their uncertainty intervals were extracted. If a study reported several effect measures in subgroups of participants defined at baseline (e.g., male versus female) we extracted and used the effect estimates separately for each subgroup. Data extraction was done by one investigator (A.A) and reviewed by another (G.D.).

We developed a set of quality criteria with binary scores to reflect (i) outcome ascertainment using pedometers or a validated questionnaire, (ii) objective reporting of exposure to mass media outlets, and (iii) large potential for confounding or selection bias. We assigned an additional two points for randomized trials with comparison groups and one point for observational studies with repeated measures.

### Statistical analysis

The reported odds ratios and difference measures were converted to relative risks (RR) using RR = OR/((1-P_o_)+(P_o_*OR)) where OR is odds ratio and P_o_ is prevalence of the outcome in the unexposed [12]. We only pooled the results in a meta-analysis if three or more studies had reported the same effect measure which was the case for ‘reduction in sedentary lifestyle’ , ‘sufficient walking’ and ‘sufficient overall physical activity’. Sufficient walking was mostly defined as moderate intensity or brisk walking for at least 150 minutes per week. Sufficient physical activity was defined as aerobic and muscle-strengthening activity lasting at least 150 minutes per week in moderate intensity (3.0 to 6.0 Metabolic Equivalent Tasks) or 60 minutes per week if vigorous.

Pooled effect measures and 95% confidence intervals were estimated using random effects models [13], with Forest plots for graphical visualization. Random effects models do not assume that included studies are estimating the exact same parameters, allowing for some variability. Heterogeneity was formally assessed with the Q and I^2^ statistics. We used meta-regression to explore heterogeneity due to study design (cohort versus before-after studies), mean age and BMI of participants and proportion of men at baseline, year of study baseline, median duration of follow-up, type of media, coverage rate, approach of intervention, and study quality.

Publication bias was evaluated with Begg’s and Egger’s tests and influence analysis was conducted to ascertain the effects of omitting individual studies. We conducted a set of alternative analyses to evaluate the sensitivity of our results to the inclusion of studies that reported the effect of mass media campaign on a slightly different outcome measure or for a different duration of campaign or follow-up. A two-sided p value of 0.05 or less was considered as significant. All statistical analyses were conducted using Stata version 11 (College Station, TX).

## Results

**Figure 1:**
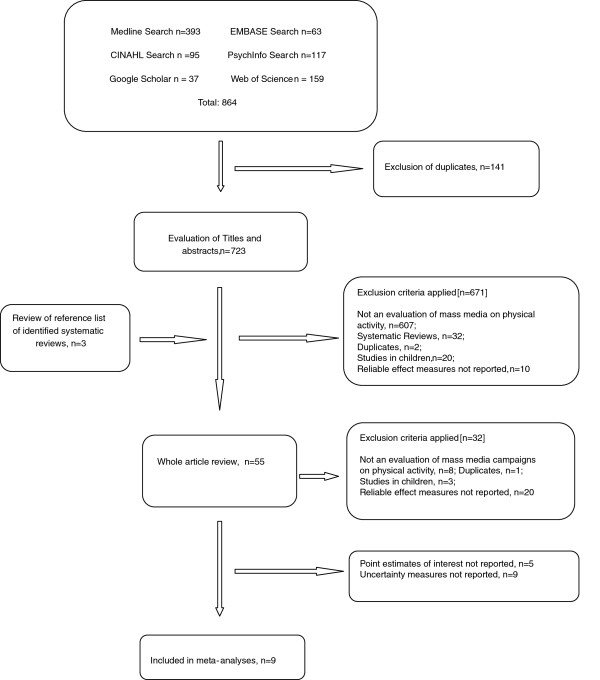
Flow-chart of study selection.

We identified 723 unique articles from the database searches and after screening the abstracts, reviewed 55 full-text articles. Of these, nine studies were selected for the meta-analysis [14-22] (Figure 1). Selected studies were all conducted in high-income countries and between 1996 and 2008. The nine studies enrolled a total of 27,601 participants and used media campaigns that lasted anywhere between 8 weeks to 3 years. Two studies used a prospective cohort design [17,18], five used a before-after design with comparison groups [14,19-22] and the remaining two used a before-after design without a comparison group [15,16]. The media campaigns were conducted on local, regional or national levels with coverage ranging from 11 to 90%. Some studies objectively reported the intensity of the mass media campaigns using ‘gross rating points’ or other similar measures [14,19-22]. Quality scores varied from 2 to 4 out of 5 points, with a median of 3 (Table 2).

**Table 2:** Characteristics of the nine selected studies

**Author (Year)**	**Bauman (A) (2003)**[14]	**Bauman (B) (2003)**[15]	**Craig (2007)**[16]	**Hillsdon (2001)**[17]	**Hopman-Rock (2005)**[18]	**Lorentzen (2007)**[19]	**Reger-Nash (2005)**[20]	**Reger-Nash (2006)**[22]	**Reger-Nash (2008)**[21]
**Country**	Australia	New Zealand	Canada	UK	Netherlands	Norway	US	US	US
**Campaign name**	Active Australia Campaign	Push Play Campaign	Canada On the Move	Active For Life	Nether-lands On the Move	Romsas in Motion	Wheeling Walks	BC Walks	WV Walks
**Campaign year**	1997-1999	1999-2000	2004	1996-1998	2000	2000-2003	2001-2002	2003	2005
**Evaluation design**	Before-After, comparing the state with the rest of Australia	Before-After	Before-After	Cohort	Cohort	Before-After Study, with comparison town	Before-after surveys: with comparison town	Before-after surveys Broome County compared to Chautauqua	Before-after: NC West Virginia compared to Cabell County
**Target population; Mean BMI**	18-75 year olds;	≥18 year olds;	18+ year olds; 28.5 kg/m^2^	16-74 year olds;	≥35 year olds;	30 - 67 year olds; 27.1 kg/m^2^	50-65 year olds, sedentary at baseline; 28.0 kg/m^2^	40-65 year olds, insufficiently active adults; 28.9 kg/m^2^	40-65 year olds, insufficiently active adults
**Overall sample Size**	5015	665	9935	3189	988	2644	1472	949	1834
**Intervention sample size**	2009	NR	NR	NR	NR	NR	719	575	1223
**Control sample size**	3006	NR	NR	NR	NR	NR	753	374	611
**Age (mean/ median, in years)**	44.4	45.9	42.5	47.2	56.0	49.0	57.0	52.4	NR
**Proportion of males, %**	44.3	45.9	33	42.5	31	44.5	33	31	33
**Duration of intervention**	8 weeks	24 months (in phases)	12 months	3 years	7 months	18 weeks in phases	12 months	8 weeks	8 weeks
**Duration of follow-up**	2 years	3 years	1 year	3 years	7 months	3 years	2 years	8 weeks	8 weeks
**Scope**	Statewide	National	National	National	National	Local	Local	Local	Regional
**Coverage**	65%	61-88%	11-31%	38%	54%	46%	90%	78%	87%
**Campaign description**	200 showings of TV ads; 6 weeks of print ads in newspapers, magazines, mail-outs to professionals, counseling kits, marketing of campaign merchandise; Campaign approach was to make physical activity (PA) a social norm	TV, Radio, Print and Outdoor; National Push Play Day was implemented; Campaign approach was to make physical activity (PA) a social norm	Electronic & Print; 2 million free pedometers distributed free; Campaign approach was to provide incentives for PA	TV & Print Magazine ads. Road-shows, workplace promotions, competitions, media advocacy; Campaign approach was for celebrities to encourage PA in the population	TV Show broadcast for 15 mins twice daily on week-days; Campaign approach was the use of risk message and to make PA a social norm	Outdoor ads especially leaflets, local meetings, stands, local television, radio, newspapers, posters. Campaign approach was to make PA a social norm	1164 TV ads, and 14 quarter-page newspaper ads; radio and cable TV as well Others: a worksite walking challenge, website, physician prescriptions, community presentations about the health benefits of walking Campaign approach was to make PA a social norm	953 TV ads, 1645 radio ads, 10 quarter-page ads in newspapers, 1314 cable TV ads. Participants encouraged to log their PA on a website; was to make PA a social norm	356 TV ads, 1763 radio ads, 39 1/8 page newspaper ads, 4256 cable TV ads Intergenerational walk to foster social networks for regular walking; Residents to log their PA on web site; Campaign approach was to make PA a social norm
**Quality Score (out of 5)**	4	2	3	2	2	2	4	4	4

NR: Not reported

**Figure 2:**
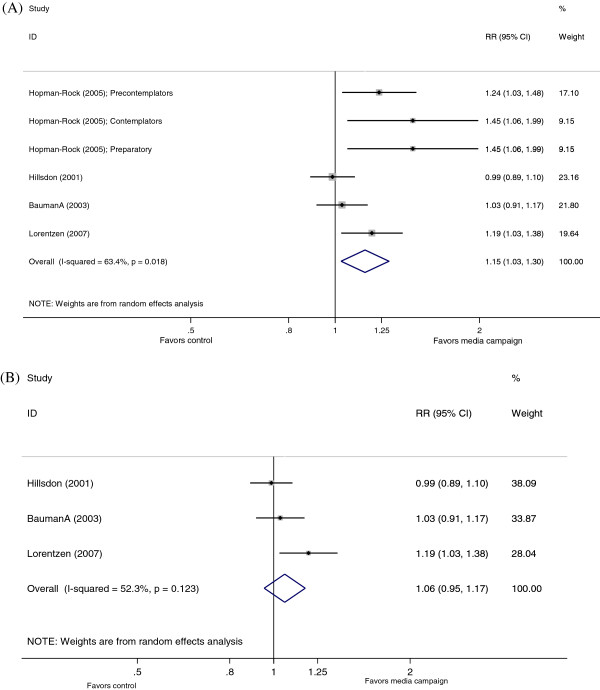
**Forest plot showing the effect of mass media campaigns on sedentary lifestyles (A) before and (B) after excluding a study with very low quality score **[18]**.**

The selected studies had reported on eleven different measures of physical activity. Four studies reported effects on reducing sedentary behavior [14,17-19]. The pooled relative risk from the six comparisons reported in these four studies was 1.15 (95% confidence interval (CI) 1.03 to 1.30). There was marked heterogeneity across these studies (I^2^=63%, p=0.018, see Figure 2 Panel A) and when a relatively low-quality study with 3 sub-groups reported was excluded, the pooled relative risk was not significantly different from the null (pooled RR 1.06, 95%CI: 0.95 to 1.17) (Figure 2 Panel B). Mean age at baseline was an important determinant of heterogeneity (p=0.054) in meta-regression analyses (Table 3). Each additional 10 years of mean age was associated with a 27% higher reduction in sedentary behavior. Media campaigns based on ‘social norm’ [18] were more likely to lead to reduction in sedentary behavior (RR=1.33, 95% CI: 1.01 to 1.43) compared with those using celebrities or based on a 'risk message' (RR=1.05, 95% CI: 0.92 to 1.21).

**Table 3:** P-values for meta-regression analyses by study characteristics

**Variable**	**Reduction in sedentary lifestyle (6 comparisons)**	**Sufficient physical activity (4 comparisons)**
Mean age	0.054	0.326
Sex	0.104	0.885
Medium	0.159	0.171
Approach^a^	0.033	0.813
Whether ads were paid	-^b^	0.970
Percent coverage	0.697	0.449
Scope	0.712	0.759
Duration of intervention	0.718	0.707
Duration of follow-up	0.552	0.297
Quality	0.119	0.447

^a^ Campaigns based on ‘social norms’ versus those using either celebrities or a risk message.

^b^ There were not enough observations for this meta-regression.

**Figure 3:**
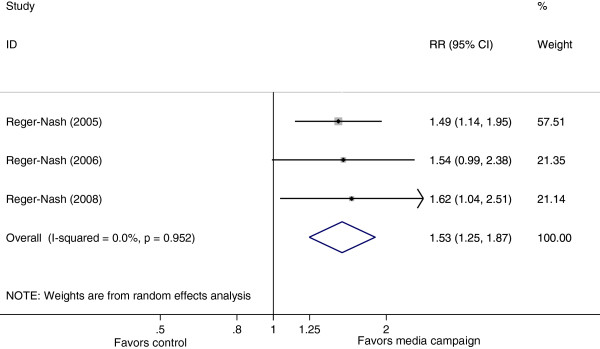
Forest plot showing the effect of mass media campaigns on sufficient walking.

Three studies [20-22] reported effects of media campaigns on achieving sufficient walking (Figure 3). When pooled, the results indicated that mass media campaigns increased the likelihood of achieving sufficient walking by 53% (RR=1.53, 95% CI: 1.25 to 1.87). There was no heterogeneity possibly because the studies were similar in design and implementation (I^2^ =0%, p=0.952).

**Figure 4:**
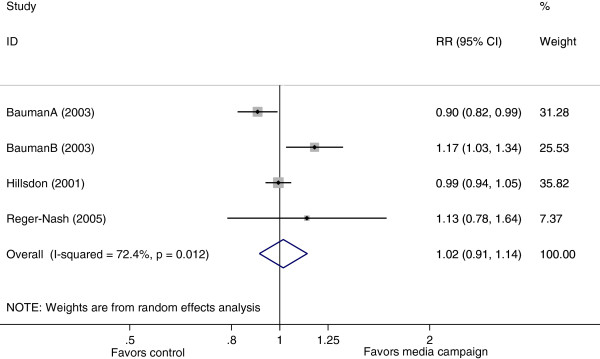
Forest plot showing the effect of mass media campaigns on sufficient physical activity.

Finally, four studies [14,15,17,20] reported the effects on achieving sufficient overall physical activity (Figure 4). The pooled RR was 1.02 (95% CI: 0.91 to 1.14) with significant heterogeneity (I^2^=72%, p=0.012). None of the selected factors for meta-regression analysis were significant sources of heterogeneity across these four studies (Table 3).

### Publication bias

Begg's and Egger's tests were suggestive of publication bias in the meta-analysis of the effect of mass media campaign on sedentary behavior (p=0.009). There was no evidence of publication bias in the other two meta-analyses: the p-value for the Egger’s test was 0.224 for sufficient walking and 0.597 for sufficient physical activity. Our influence analysis showed that the pooled effect estimates were not dominated by any of the individual studies on sufficient walking or sufficient overall physical activity (results not shown).

### Other sensitivity analyses

When we included estimates from a study [16] that had a more stringent definition for sufficient walking (1 hour per day as opposed to 150 minutes per week) the pooled RR decreased from 1.53 (95% CI: 1.25 to 1.87) to 1.35 (95% CI: 1.05 to 1.72). There was substantial heterogeneity in the estimates (I^2^=68%, p=0.024) but none of the selected factors was a significant determinant of heterogeneity in meta-regression analyses. Furthermore, one of the three studies on sufficient walking [20] also reported the effects using a longer follow-up time (12 months compared with 3 months in other studies). We substituted the 12-month effect estimate from this same study for the 3-month effect in a sensitivity analysis. The pooled RR and heterogeneity did not change materially (RR=1.50, 95% CI: 1.20 to 1.88 (I^2^=0.0%, p=0.901)).

The pooled estimates for sufficient physical activity did not change materially when we excluded one study for which the duration of intervention was 8 weeks compared to the others with interventions lasting up to 24 months (RR = 1.06; 95% CI: 0.97 to 1.53). Heterogeneity remained substantial (I^2^=63.5%, p=0.065) but none of the selected factors was a significant determinant of heterogeneity.

## Discussion

Our meta-analyses of nine prospective studies found that mass media campaigns may improve sufficient walking but may not reduce sedentary lifestyles or encourage participants to achieve recommended levels of overall physical activity. We included moderate and high quality studies and our results for walking and sufficient physical activity were robust to the selected sensitivity analyses whereas the results for sedentary behavior were influenced by a single study and showed evidence of publication bias. Except for the ‘approach’ of the campaign and the mean age of participants that seemed to change the effect of campaigns on sedentary lifestyle, we found no other significant determinant of heterogeneity among the selected studies.

Our results have important implications for the design and implementation of NCD prevention programs that aim to improve physical activity. The pooled RRs suggest that a well-designed mass media campaign may increase the likelihood of achieving sufficient walking by 53% which is equivalent to about 80 minutes per week. A recent meta-analysis of prospective studies found that an additional 150 minutes of walking over 5 days led to a 19% reduction in risk of coronary heart disease [23]. Applying this effect size to our results indicates a potential 11% reduction in risk of coronary heart disease following a well-designed mass media campaign.

Results from four previous systematic reviews of mass media campaigns and physical activity were mixed. The investigators qualitatively assessed the totality of evidence but did not conduct a meta-analysis. Two previous reviews concluded that mass media have either no effect or a very small effect on physical activity [8,9] and another review suggested a significant effect on physical activity levels without specifying the effect size or the type of activities that were influenced [7]. The original studies included in these four reviews reported different outcome measures and used widely different evaluation methods including sub-optimal designs such as post-campaign cross-sectional surveys [7,8]. Systematic reviews of mass media and other health behaviors have faced a similar challenge. For instance, mass media interventions were found effective in encouraging their audience to quit smoking, but the effects were derived from heterogeneous studies of variable quality [24,25]. Similarly, pooled analyses of mass media and diet found a beneficial effect but the pooled studies were widely different in design and quality [26-28].

Other interventions to promote physical activity have been systematically reviewed. Several prior meta-analyses have reported the effects of pedometers [3], internet-based interventions [4,5] and telephone calls [6] on physical activity. The pooled effects were generally larger than those we observed for media campaigns, but similar to those reported for exercise referral schemes [29] and computer-tailored interventions [3,30].

### Strengths and limitations

We selected 9 moderate to high-quality studies and extracted comparable metrics of effect. When comparable metrics were not reported, we used the reported results to calculate a common metric for pooling. We explored the sources of heterogeneity across studies using meta-regression. However, our systematic review was still limited by the marked differences in the reported outcomes of the selected studies. We did not have sufficient power to detect differences across studies by study-level characteristics due to the small number of selected studies.

We were also unable to evaluate the dose–response curve for mass media campaigns because most studies did not provide sufficient information to calculate a uniform metric for exposure to campaign (such as ‘gross rating points’). Measurement error in assessment of physical activity is possible in the original studies because few studies used validated questionnaires or objective measurements of activity. This study was also limited by its focus on adults and the findings among young people may differ from ours. Finally, the selected studies were all conducted in developed countries. Therefore, our results cannot be generalized to developing countries.

## Conclusions

In summary, our results indicated that mass media campaigns may promote walking, but may not reduce sedentary behavior or lead to achieving sufficient physical activity. Further research is required to examine the effect of mass media campaigns on other measures and types of physical activity (such as time spent walking and overall time spent in physical activity). We suggest that investigators report intensity and frequency of mass media campaigns using standard metrics and measure physical activity objectively or using validated questionnaires. Similar evaluations are needed to examine the effect of mass media campaigns in low and middle-income countries and in different cultural milieus.

## Competing interest

The authors declare that they have no competing interests.

## Authors’ contributions

GD designed the study. AA and KH conducted the search and screened the articles. AA extracted the data and conducted the analyses. GD and AA wrote the first draft of the manuscript. KH provided comments on the manuscript draft. GD oversaw the process and is the study guarantor. All authors read and approved the final manuscript.
